# Association of lifelong exposure to cognitive reserve-enhancing factors with dementia risk: A community-based cohort study

**DOI:** 10.1371/journal.pmed.1002251

**Published:** 2017-03-14

**Authors:** Hui-Xin Wang, Stuart W. S. MacDonald, Serhiy Dekhtyar, Laura Fratiglioni

**Affiliations:** 1 College of Public Health, Zhengzhou University, Zhengzhou, China; 2 Aging Research Center, Department of Neurobiology, Caring Sciences and Society, Karolinska Institutet and Stockholm University, Stockholm, Sweden; 3 Stress Research Institute, Stockholm University, Stockholm, Sweden; 4 Department of Psychology, University of Victoria, Victoria, British Columbia, Canada; 5 Institute on Aging and Lifelong Health, University of Victoria, Victoria, British Columbia, Canada; 6 Department of Clinical Neuroscience, Division of Psychology, Karolinska Institutet, Stockholm, Sweden; 7 Stockholm Gerontology Research Center, Stockholm, Sweden; University of California San Francisco Memory and Aging Center, UNITED STATES

## Abstract

**Background:**

Variation in the clinical manifestation of dementia has been associated with differences in cognitive reserve, although less is known about the cumulative effects of exposure to cognitive reserve factors over the life course. We examined the association of cognitive reserve-related factors over the lifespan with the risk of dementia in a community-based cohort of older adults.

**Methods and findings:**

Information on early-life education, socioeconomic status, work complexity at age 20, midlife occupation attainment, and late-life leisure activities was collected in a cohort of dementia-free community dwellers aged 75+ y residing in the Kungsholmen district of Stockholm, Sweden, in 1987–1989. The cohort was followed up to 9 y (until 1996) to detect incident dementia cases. To exclude preclinical phases of disease, participants who developed dementia at the first follow-up examination 3 y after the baseline were excluded (*n* = 602 after exclusions). Structural equation modelling was used to generate latent factors of cognitive reserve from three periods over the life course: early (before 20 y), adulthood (around 30–55 y), and late life (75 y and older). The correlation between early- and adult-life latent factors was strong (γ = 0.9), whereas early–late (γ = 0.27) and adult–late (γ = 0.16) latent factor correlations were weak. One hundred forty-eight participants developed dementia during follow-up, and 454 remained dementia-free. The relative risk (RR) of dementia was estimated using Cox models with life-course cognitive reserve-enhancing factors modelled separately and simultaneously to assess direct and indirect effects. The analysis was repeated among carriers and noncarriers of the apolipoprotein E (APOE) ε4 allele. A reduced risk of dementia was associated with early- (RR 0.57; 95% CI 0.36–0.90), adult- (RR 0.60; 95% CI 0.42–0.87), and late-life (RR 0.52; 95% CI 0.37–0.73) reserve-enhancing latent factors in separate multivariable Cox models. In a mutually adjusted model, which may have been imprecisely estimated because of strong correlation between early- and adult-life factors, the late-life factor preserved its association (RR 0.65; 95% CI 0.45–0.94), whereas the effect of midlife (RR 0.73; 95% CI 0.50–1.06) and early-life factors (RR 0.76; 95% CI 0.47–1.23) on the risk of dementia was attenuated. The risk declined progressively with cumulative exposure to reserve-enhancing latent factors, and having high scores on cognitive reserve-enhancing composite factors in all three periods over the life course was associated with the lowest risk of dementia (RR 0.40; 95% CI 0.20–0.81). Similar associations were detected among APOE ε4 allele carriers and noncarriers. Limitations include measurement error and nonresponse, with both biases likely favouring the null. Strong correlation between early- and adult-life latent factors may have led to a loss in precision when estimating mutually adjusted effects of all periods.

**Conclusions:**

In this study, cumulative exposure to reserve-enhancing factors over the lifespan was associated with reduced risk of dementia in late life, even among individuals with genetic predisposition.

## Abstract 2

In a community-based cohort study, Serhiy Dekhtyar and colleagues examine the association between engagement in various cognitive reserve-enhancing factors at early, mid, and late life and risk of dementia concurrence after 75.

## Author summary

**Why was this study done?:**

- It has emerged from previous literature that lifestyle factors such as physical exercise, intellectual stimulation, or leisure activities are associated with a reduced risk of dementia occurrence in late life. One possibility is that these activities provide protection in the form of reserve, facilitating the maintenance of cognitive function in the face of cumulative brain damage.
- It is, however, still largely unknown how the risk of dementia is shaped by various reserve-stimulating lifestyle factors simultaneously taking place throughout the entire life course.
- Our study was designed to examine the association between the risk of dementia occurrence after age 75 and engagement in a variety of reserve-enhancing activities over the entire life course.

**What did the researchers do and find?:**

- We estimated the risk of dementia occurrence in a cohort of individuals aged 75 y and older conditional on their engagement in ten activities that were expected to promote reserve in three stages over the life course.
- We found that, on its own, engagement in early-, adulthood-, and late-life reserve-enhancing activities was associated with a reduced risk of dementia. However, when all three factors were simultaneously evaluated for their association with dementia, the effects of early-life and adulthood factors were attenuated. This could be due to the strong correlation between early-life and adulthood factors, whereas the late-life factor was only moderately related to either early-life or adulthood markers of reserve.
- An important finding was that increased frequency of engagement in stimulating activities over the life course was associated with a progressively reduced risk of dementia, suggesting a dose-response effect between life-course reserve and dementia risk. This effect appeared to operate irrespective of genetic predisposition to dementia.

**What do these findings mean?:**

- These findings strongly suggest that development of dementia is a lifelong process that begins decades before the onset of the disease.
- At the same time, it is never too late to initiate interventions aimed at risk modifying, since late-life engagement in stimulating activities was associated with a lower risk of dementia.
- However, because reserve-enhancing activities are correlated over the life course, and because the lowest risk is enjoyed by individuals with increased frequency of stimulating engagements, the most effective strategies are likely to be those emphasizing risk reduction throughout the entire life course.

## Introduction

The relevance of cognitive reserve-enhancing factors as contributors to dementia risk has emerged from several longitudinal population-based studies [1–3] and has been confirmed by pathological and clinical data [4]. Thus, numerous studies have reported an increased risk of dementia in less educated persons [5,6]. Higher education could help build cognitive reserve—a set of skills or repertoires that increase an individual’s ability to cope with dementia pathology later in life [3,7]. Some studies have suggested that childhood socioeconomic status (SES) may be of importance for the development of dementia in late life [8,9]. A poor-quality environment during childhood or adolescence may prevent the brain from reaching full levels of maturation, leading to low cognitive reserve, which in turn could put people at higher risk for dementia. On the other hand, few years of schooling could be a marker of cognitive abilities that may have both gestational and genetic origin [10]. In any case, the first decade of life appears to be a critical period for developing dementia later in life [11,12].

In addition to early life contributors, the functional efficiency of cognitive networks may be promoted by adulthood factors, such as occupational attainment or leisure activities, leading to higher cognitive reserve. There is growing support for the hypothesis that mental stimulation in middle age (e.g., mental occupational demands [13] and work complexity [14–16]), as well as in old age (e.g., leisure activities [1,17]), may reduce the risk of dementia. Biologically, mental stimulation could selectively increase synaptogenesis in adulthood, whereas physical exercise might enhance non-neuronal components of the brain, such as vasculature [18]. The ability of the adult brain to respond to environmental stimuli by activating compensatory processes, thus, could be sustained in late life [19]. Therefore, it appears that factors associated with dementia risk originate in different periods throughout the entire life course, including early, adult, and late life.

Few studies have been able to examine the effects of cumulative exposure to cognitive reserve-enhancing factors across the life span. Exposure to these factors during different life periods may act alone or combine in clusters affecting health in later life [20,21]. It has been suggested that dementia is not determined at a single time period but rather results from a complex interplay between genetic and environmental exposures throughout the life course [22,23]. Although life-course models for late-onset diseases have received increased attention in the epidemiological field [21], this approach has not yet been widely applied to dementia [22], with the exception of a few studies [12,24,25]. Using a life-course approach, this study aims to test the hypotheses that (1) cognitive reserve-related factors operating at various life periods each are potentially associated with decreased risk of the occurrence of dementia, (2) cumulative exposure to reserve-related factors is associated with a progressively reduced risk of dementia, and (3) the risk of dementia later in life is influenced by the interaction between lifelong exposure to cognitive reserve-related factors and genetic factors (e.g., apolipoprotein E [APOE] ε4).

## Methods

### Ethical approval

All phases of the project received approval from the Ethics Committee at Karolinska Institutet (Dnrs: 87:234; 90:251; 94:122; 97:413; 99:308; 99:025; 01:020). All individuals participating in the study completed a written informed consent form as stipulated in the ethical approval. For those participants who became cognitively impaired over time, consent was obtained from the next of kin.

### Study population

The study population is derived from the Kungsholmen Project, a longitudinal community-based study that included all inhabitants of the Kungsholmen district in Stockholm, Sweden, aged 75 y and older on 1 October 1987 (*n* = 2,368) [26,27]. Of the 1,810 baseline participants, 1,473 were diagnosed without dementia by the two-phased design. These participants were approached again every 3 y for first, second, and third follow-up examinations. Because impaired cognition or institutionalization may limit participants’ current activity [28], we excluded 98 individuals with a baseline Mini-Mental State Examination (MMSE) score of less than 23, as well as those residing in an institution. During the first follow-up examination 3 y after the baseline, 441 of the baseline participants could not be clinically examined (269 died, and 172 refused participation), whereas 158 individuals were diagnosed with dementia and were excluded from the study to avoid the possibility that they may already have been in the preclinical phase of dementia when interviewed at baseline [29]. Another 44 participants who refused the second follow-up examination were also excluded. The resulting population eligible for analysis consisted of 732 individuals without dementia. Of these, we removed individuals with missing information on education (*n* = 3) and occupation (*n* = 109), as well as 18 women who never entered the labour market, resulting in 602 dementia-free participants at both baseline and the first 3-y follow-up who were followed for up to 9 y to detect incident dementia cases.

### Dementia diagnosis

The clinical diagnosis of dementia was obtained in accordance with the third revised Diagnostic and Statistical Manual of Mental Disorders (DSM-III-R), with some modification [30]. A two-phased diagnostic procedure [27] was employed, whereby two physicians working independently made a preliminary diagnosis, and a third opinion was sought in the event of discordant assessments by the original examiners. Modifications to the DSM-III-R criteria included adding a diagnosis of questionable dementia for individuals with evident impairment in all requisite functions except one and defining cognitive impairment on the basis of objective neuropsychological assessment [31]. In the current study, only clinically definite cases of dementia were included. The time of dementia onset was assumed to be the midpoint between two examinations or the midpoint between examination and the date of death due to dementia.

### Assessment of life-course cognitive reserve factors

We assessed cognitive reserve-related indicators from three distinct periods of the life course: early life, adult life, and late life.

Early life cognitive reserve-related factors (before 20 y) included the following:

- Educational attainment collected from participants at the study baseline (1987–1989) as a result of the structured questionnaire administered by one of the two trained nurses (S2 Text). The highest degree achieved was recorded and subsequently categorized as elementary, professional, intermediate school, high school, or university.
- Early-life SES measured by the number of siblings grown up with and substantive work complexity at 20 y. A trained nurse recorded the number of siblings during the same baseline interview in which educational attainment was assessed. We expected that a larger number of siblings would be a proxy for lower SES, since research has shown that around the time of this study population’s birth (1885–1912), elite socioeconomic groups had started limiting their fertility, whereas the less privileged strata did not begin this transition until several decades later [32]. Substantive work complexity at age 20 was collected at an informant interview during the first follow-up examination (1990–1991; S2 Text) that aimed to retrospectively explore lifespan work activities, by inquiring about the employer, job title, time period, and tasks at all jobs lasting at least 6 mo [33]. Substantive work complexity at 20 y was recorded in accordance with the work complexity matrix [34] as reported below.

Adult life cognitive reserve-related factors (around 35–55 y) included the following:

- Complexity of work with data and people for the longest-held occupation in adult life. Work complexity scores were recorded in accordance with a work complexity matrix [34] that was based on the estimation of more than 12,000 occupations rated during on-site occupational assessments in the United States. Occupational categories of the 1980 Swedish census were matched to the best-fitting category in the 1970 US census [14]. The measures for each occupation were created to reflect the levels of complexity at which a worker in a particular occupation functions according to four dimensions: substantive complexity of work (score range 0–10) and complexity of work with data (0–6), with people (0–8), and with things (0–7), with higher scores indicating greater complexity. Work complexity with things was excluded since it was not found to affect dementia in a previous study using the same material [16] and because of its weak contribution to the latent variable. A large sample study assessing inter-rater agreement of the complexity ratings of different occupations yielded reliability estimates: 0.85 for complexity of work with data and 0.87 for complexity of work with people [35].
- Job demands and decision latitude for the longest-held occupation in adult life. Job demands designate the use of skills to perform job tasks, whereas decision latitude indicates the extent of decision authority at a workplace [36]. Both measures were derived in accordance with a psychosocial job exposure matrix [37] for the longest occupational period. If participants reported spending the longest portion of their life working inside the household, their second-longest occupation was used. A high score indicates a higher level of psychosocial job demands and decision latitude. The scores ranged from 2.5 to 9.0 for job demands and 2.2 to 8.6 for control at work. Job demand and job controls derived from the job exposure matrix have been shown to correlate with self-reported levels among the same individuals (average r = 0.6) in a validation study [38]. Information on occupational attainment was collected from informants (relatives or other knowledgeable person) at the first follow-up examination (1990–1991) during the interview on lifetime work history. Interviews were conducted by one of the two nurses specially trained in occupational coding. Informants were used for individuals with dementia as well as dementia-free participants (S2 Text). The interview questionnaire was developed by an expert in occupational medicine and aimed to explore the lifespan work activities of all jobs lasting at least 6 mo. Substantive complexity ratings were added to occupations at 20 y, whereas measures of demands, decision latitude, and complexity of work with data and with people were linked with the longest-held job.

Late-life cognitive reserve-related factors (75 y and older) included the following:

- Late-life leisure activities. Information on leisure activities was obtained by means of a personal interview conducted by trained nurses at baseline (1987–1989) (S2 Text). Participants were asked whether they regularly engaged in activities and what those specific activities were, resulting in 29 being identified. A mental, social, and physical component score was assigned to each of these activities, with grading of the three components defined as follows: 0 = none, 1 = low, 2 = moderate, and 3 = high. The sum of the component scores, which had a range of 0–18, was calculated based on the grading for each of the three activities [39]. To validate the scoring, 13 cognitively intact raters, aged 75 y or older, were asked to individually complete a questionnaire containing a list of the 29 activities together with scoring instructions. Reliability analyses revealed a satisfactory result: values for Cronbach’s α were 0.89 for the mental component, 0.95 for the physical component, and 0.82 for the social component.

### Covariates

All covariates were assessed at the study baseline (1987–1989). In addition to age and gender (both extracted from the National Population Register), baseline cognitive functioning was evaluated using the MMSE, with a score of 30 indicating unimpaired performance. Depression was assessed through self-reported symptoms such as feeling lonely and constantly being in a bad mood. Comorbidities were ascertained by reviewing the hospital discharge diagnoses through the Stockholm computerized inpatient register system with coverage since 1969. Based on the International Classification of Disease, 8th edition (ICD-8) [40], we identified coronary heart disease (ICD-8: 410–414), cerebrovascular disease (ICD-8: 430–438), diabetes mellitus (ICD-8: 250), malignancy (ICD-8: 140–208 and 230–239), and hip fracture (ICD-8: 820). Comorbidity was defined as the presence of any of these conditions. Genomic DNA was prepared from peripheral blood samples at baseline, and APOE genotyping using a standard polymerase chain reaction procedure [41] was performed by two technicians who were blind to all other data.

### Statistical analysis

Logistic regression was used to examine differences between participants and nonparticipants. Structural equation models (SEMs) were computed to derive the best-fitting measurement model for ten individual lifelong reserve-enhancing indicators. Using various fit criteria [42,43], three separate latent reserve measures were generated, comprised of early-, adult-, and late-life cognitive reserve-enhancing composite indicators. Cox regression models (age- and sex-adjusted) were then used to estimate the hazard ratio (HR) and 95% confidence intervals of dementia occurrence for each of the three latent composite reserve-enhancing factors. In an additional set of analyses, we further adjusted for late-life cognitive functioning as well as the presence of comorbidity and depressive symptoms. The three latent composite factors were analysed as continuous variables, quartile categories, and dichotomized variables contrasting the top three quartiles versus the lowest one. First, each composite indicator for a specific life period was analysed separately, and then all three were entered into the same model to verify their independent effects.

We decomposed the total effect of early-, adult-, and late-life composite factors on dementia risk. First, a full model including all three life-course factors and covariates was fit, with estimated parameters producing the direct effects of early-, adult-, and late-life factors. Next, a series of reduced models that included only early-, adult-, or late-life indicators was estimated, with parameters from these models producing the total effect of each life-course indicator. The difference between the total and the direct effect for each of the latent life-course factors yielded an estimate of its indirect effect through all mediating factors. The significance of the indirect effect was tested through the model likelihood ratio [44].

As the risk of dementia may be affected by an interaction between genetic and environmental factors [45], formal tests of statistical interactions between the life-course cognitive reserve-enhancing composite factors and APOE ε4 status were performed by introducing an interaction term. To further assess the possible interaction between genetic predisposition and cognitive reserve, we estimated the effects of reserve-enhancing composite factors among both APOE ε4 allele carriers and noncarriers.

## Results

Logistic regression showed that the population analysed in the study (*n* = 602) and the eligible individuals who were not included because of missing data on covariates (*n* = 130) did not differ with respect to age (odds ratio [OR] 0.98, 95% CI 0.94–1.03), sex (OR 0.92, 95% CI 0.58–1.40), presence of depression (OR 1.16, 95% CI 0.76–1.77), or comorbidity (OR 1.27, 95% CI 0.89–1.81). Table 1 shows the characteristics of study participants 4–9 y before dementia diagnosis. As expected, individuals who would develop dementia later were relatively older, more likely to be female, had more depressive symptoms, reported poorer cognitive functioning, and were more likely to be APOE ε4 allele carriers.

**Table 1:** Baseline characteristics of the study population including 454 persons who remained free of dementia during the follow-up and the 148 individuals who developed dementia over an average of 6 y of the follow-up.

	Dementia-free (*n* = 454)	Incident dementia (*n* = 148)	*p*-Value
Age (years), mean ± SD	80.2 ± 4.4	81.1 ± 4.2	0.03
MMSE score (0–30), mean ± SD	27.6 ± 1.4	27.3 ± 1.3	0.04
Women, %	72.7	79.7	0.09
Depressive symptoms, %	24.7	33.8	0.03
Comorbidity, %	22.2	28.4	0.13
APOE ε4 allele, %	23.1	32.4	0.03

APOE, apolipoprotein E; MMSE, Mini-Mental State Examination; SD, standard deviation.

P—values are from T-test for continuous variables or χ^2^ test for categorical variables.

**Fig 1:**
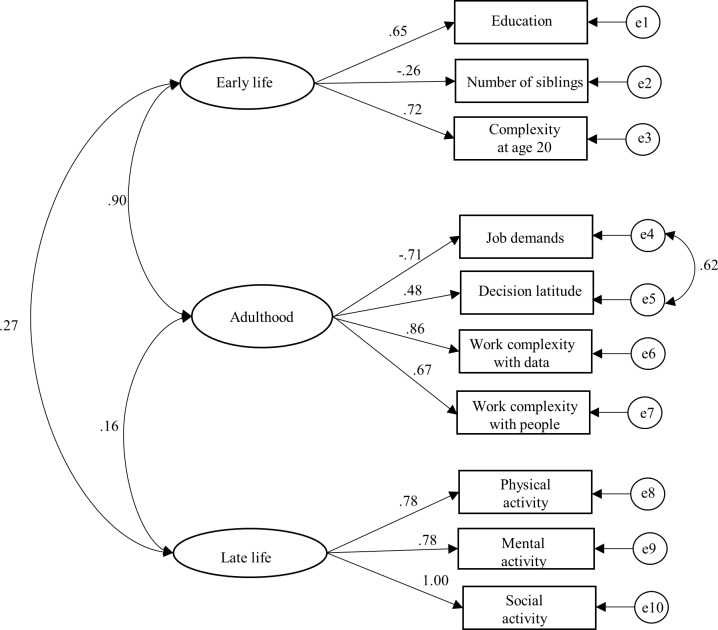
Standardized estimates from the structural equation model (SEM) for the three composite factors and corresponding cognitive reserve indicators from three life periods (early life, adulthood, and late life). SEM fit statistics: χ^2^ = 99.81, df = 31, *p* < 0.001; χ^2^/df ratio = 3.22; goodness-of-fit index (GFI) = 0.967; comparative fit index (CFI) = 0.901; and root mean square error of approximation (RMSEA) = 0.061.

The best-fitting latent measurement model for the three life-course cognitive reserve-enhancing composite factors (early, adult, and later life) is presented in Fig 1. For each latent factor, an individual’s factor score was estimated by (1) standardizing measurements on the raw indicators, (2) multiplying the standardized score for each indicator by its corresponding SEM factor-score weight, and (3) summing the products to yield three separate latent factor-score estimates for the early-, adult-, and late-life periods (for more details, see S1 Text). The distribution of the latent variables was as follows: early life (range from −1 to 1, mean = 0.02, and standard deviation [SD] = 0.491), adult life (range from −1 to 1, mean = 0.02, and SD = 0.372), and late life (range from −1 to 1, mean = 0.10, and SD = 0.655). Relative risks from models with latent variables should be interpreted as per a 1 unit SD increase in the underlying latent factor. The correlation between the early- and the adult-life latent factors was strong (γ = 0.9).

We began by analysing the association between the risk of dementia occurrence and continuous cognitive reserve-enhancing composite factors derived from the SEM model on the risk of dementia (S1 Table). In separate minimally adjusted (age and sex) Cox models, a reduced risk of dementia was associated with early- (relative risk [RR] 0.61; 95% CI 0.41–0.90), adult- (RR 0.53; 95% CI 0.31–0.91), and late-life (RR 0.70; 95% CI 0.53–0.92) continuous composite factors. Estimating the association between dementia occurrence and all three life-course reserve factors in a simultaneous-entry model yielded nonsignificant risk estimates of early- (RR 1.19; 95% CI 0.22–6.39), adult- (RR 0.57; 95% CI 0.06–5.26), and late-life factors (RR 0.80; 95% CI 0.58–1.09).

We then converted the continuous reserve-enhancing factors into four categories based on the quartile distribution and assessed their associations with the risk of dementia occurrence, first in minimally adjusted separate Cox models and then in a fully adjusted model with all three factors estimated simultaneously (Table 2). In separate models, all quartile categories of the early-life reserve composite factor were associated with a reduced risk, relative to the bottom category, although only quartile four was statistically significant (relative risk [RR] 0.45; 95% CI 0.27–0.77). Similarly, all quartile categories of the adult-life reserve composite factor were associated with a reduced risk of dementia, relative to the bottom quartile (quartiles two and four were statistically significant; RR 0.57; 95% CI 0.37–0.89, and RR 0.46; 95% CI 0.27–0.77, respectively). All three quartiles of the late-life reserve composite factor were associated with a statistically reduced risk of dementia relative to the bottom quartile category. When all three composite factors were simultaneously entered into a single fully adjusted model, only the late-life composite factor (except quartile 2) preserved its statistical association with a reduced risk of dementia, whereas early- and adult-life reserve composite factors were no longer associated with a reduced risk of dementia.

**Table 2:** Number of participants, incident dementia cases, and relative risk (RR) (95% confidence interval [CI]) of dementia in relation to the cognitive reserve latent factors acting at different time periods in the life course.

	Number of participants	Number of cases	RR (95% CI)	RR (95% CI)
Reserve factors			From separate models adjusted for age and sex	From one model with full adjustment
**Early life**				
Quartile categories				
1st	149	43	1	1
2nd	152	40	0.71 (0.46–1.09)	1.03 (0.60–1.78)
3rd	151	38	0.75 (0.49–1.17)	1.15 (0.52–2.53)
4th	150	27	0.45 (0.27–0.77)	0.60 (0.17–2.17)
**Adulthood**				
Quartile categories				
1st	150	46	1	1
2nd	151	34	0.57 (0.37–0.89)	0.58 (0.34–1.00)
3rd	150	40	0.76 (0.50–1.17)	0.83 (0.39–1.74)
4th	151	28	0.46 (0.27–0.77)	0.99 (0.29–3.41)
**Late life**				
Quartile categories				
1st	150	50	1	1
2nd	151	37	0.60 (0.39–0.92)	0.68 (0.43–1.06)
3rd	151	33	0.52 (0.33–0.81)	0.62 (0.39–0.99)
4th	150	28	0.44 (0.28–0.71)	0.59 (0.35–0.99)

Full adjustment: age, sex, depressive symptoms, comorbidity, and baseline cognitive function.

**Fig 2:**
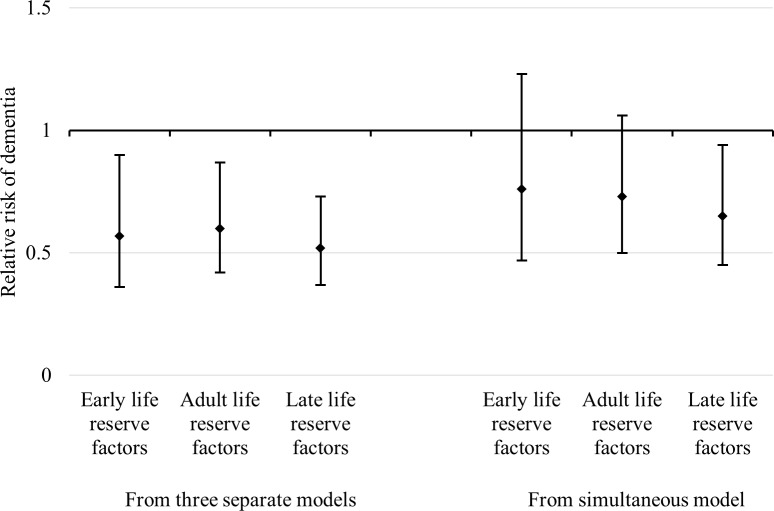
RRs and 95% CIs of dementia in relation to early-life, adulthood, and late-life cognitive reserve-enhancing composite factors. The RRs and 95% CIs on the left-hand side were estimated from three separate Cox models adjusted for age and sex. The RRs and 95% CIs on the right are from a single Cox model that simultaneously estimated the effects of composite indicators with additional adjustment for age, sex, depressive symptoms, comorbidity, and baseline cognitive function. Early-life, adulthood, and late-life cognitive reserve-enhancing composite indicators are dichotomized (top three quartiles versus the bottom quartile) based on the quartile transformation of continuous variables (factor scores) extracted from an SEM model.

Next, we converted all reserve factors into a dichotomous variable derived from a quartile distribution by combining the top three quartiles of each composite factor and contrasting them against the bottom quartile. The association between the risk of dementia occurrence and the dichotomized cognitive reserve latent factors was estimated in three separate minimally adjusted models, as well as in a single fully adjusted Cox regression (Fig 2). Whereas all three dichotomized life-course cognitive reserve-enhancing composite factors were associated with a reduced risk of dementia (early-life RR 0.57; 95% CI 0.36–0.90; adult-life RR 0.60; 95% CI 0.42–0.87; late-life RR 0.52; 95% CI 0.37–0.73) when analysed separately in minimally adjusted models, only the late-life factor remained statistically associated with a reduced risk of dementia (RR 0.65; 95% CI 0.45–0.94) in the simultaneous fully adjusted model. The association between dementia risk and the adult-life dichotomized composite factor was attenuated and remained marginally statistically significant (RR 0.73; 95% CI 0.50–1.06), whereas the early-life reserve-enhancing composite factor was the most attenuated (RR 0.76; 95% CI 0.47–1.23). In additional analyses (S2 Table), we found that 39.3% of the total association between the early-life composite factor and the hazard rate of dementia was attributed to its indirect association with adult- and late-life factors.

**Fig 3:**
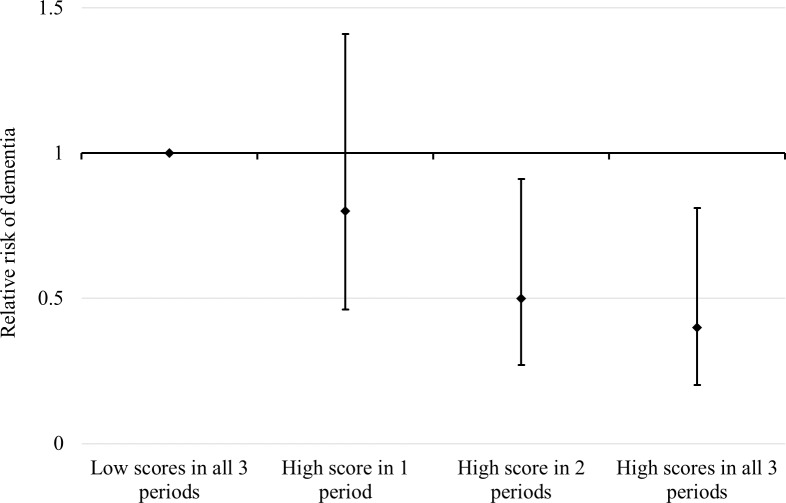
RRs and 95% CIs of dementia in relation to cumulative exposure to reserve-enhancing composite factors over the life course. Estimated from a simultaneous-entry Cox regression model adjusted for age, sex, depressive symptoms, comorbidity, and baseline cognitive function.

Early-life and adulthood latent factors were highly correlated (γ = 0.9). This implies that total effects of either factor on dementia from individually estimated models might not be independent and that the mutually adjusted effect of these variables might not be precisely estimated because of collinearity. To better understand the structure of exposure status without explicitly modelling the effect of each period, we divided participants into different groups according to their exposure status (high versus low) at the three time periods (early, adult, and late life). Because of study size limitations, we generated an index distinguishing between (1) a high-reserve level for just a single life period, (2) a high level for two out of the three life periods, and (3) a high reserve for all three life periods. We then estimated the risk of dementia for each of these three groups relative to those with a low level of reserve in all life periods (Fig 3).

Relative to individuals with low scores on all three life-course cognitive reserve-enhancing composite factors over the life course, there was no significant reduction in the risk of dementia for individuals with high scores on the composite indicator in one life period (RR 0.80; 95% CI 0.46–1.41). Having high scores on the composite indicators in two life periods was associated with a significantly reduced risk of dementia (RR 0.50; 95% CI 0.27–0.91), with high scores in all three life periods associated with an even greater dementia risk reduction (RR 0.40; 95% CI 0.20–0.81) in the model adjusted for age, sex, depressive symptoms, comorbidity, and baseline cognitive function (Fig 3).

Finally, we found no evidence of a synergistic interaction between each of the life-course cognitive reserve-enhancing composite factors and genetic predisposition (APOE ɛ4 status) on risk of dementia (formal statistical test for interaction). Stratified analysis according to APOE ɛ4 status furthermore revealed that having high cognitive reserve in two or three periods over the life course was associated with a lower risk of dementia in both ɛ4 allele carriers (RR 0.5; 95% CI 0.3–0.9) and ɛ4 allele noncarriers (RR 0.7; 95% CI 0.5–0.97).

## Discussion

Using a life-course approach, we found in our cohort that cognitive reserve-stimulating activities during early, adult, and late life were associated with a lower risk of dementia occurrence, although the early-life association was partially (39.3%) mediated by mid- and late-life activities. We observed a reverse dose-response relationship between dementia risk and increased duration of exposure to reserve-enhancing factors over the life course. However, the hypothesized interaction between cumulative exposure to cognitive reserve factors and genetic predisposition on the risk of dementia was not confirmed. The risk of dementia due to cumulative exposure to cognitive reserve factors over the life course was reduced irrespective of individuals’ genetic predisposition to dementia.

This study has several strengths. First, this prospective study collected information on exposures at least 4 y before incident dementia cases were diagnosed. Second, to avoid ascertainment bias [46], only cognitively intact community dwellers with no dementia were included, and people who developed dementia during the first 3 y were excluded from the study. Third, the three latent reserve-enhancing composite factors were estimated using structural equation modelling with a good overall fit. The use of latent factors has several advantages including (1) the incorporation of the interrelated observed measures, (2) the pooling of common variance across multiple index measures for each latent measure (increased convergent validity), and (3) the correction for unreliability in observed measures by attenuating measurement error.

Limitations of this study include possible measurement error in measuring adult-life work complexity, job demands, and decision latitude, as well as late-life leisure activities. However, since information was obtained from the same source for all participants, the misclassification is likely to be nondifferential and therefore should only lead to an underestimation of the true population effects. In addition, nonresponse bias may have occurred, but it should not have much bearing on the results because the distribution of participants and nonparticipants was largely comparable with respect to demographic and health indicators. The correlation between the early-life and the adulthood latent factors was strong (γ = 0.9). A respecified SEM model was fit, excluding occupational complexity at 20 y as an indicator of the early-life factor, in an attempt to reduce this association. While the correlation between the early and adult latent factors declined from 0.9 to 0.64, model fit deteriorated too. As one approach to circumventing collinearity concerns, we examined the general exposure structure by evaluating the effects of having high scores on latent factors over consecutive periods in life.

The results of the current study should first be considered in light of studies on cognitive reserve measures collected at single periods during the life course [5,12,23]. Thus, consistent with previous research, we have demonstrated that high scores on early-life measures of cognitive reserve [5,12,24,47,48], engagement in adult reserve-enhancing activities, such as complex occupational roles [6,12,16,24], and late-life engagement in leisure activities are all associated with a lowered risk of dementia [49]. Although reserve-enhancing activities from distinct periods of life have been individually linked with the risk of dementia previously, studies on reserve contributors spanning several periods over the life course have been lacking, with the exception of a few studies [12,24]. Notably, we extend this limited literature in an important way by adding previously unmeasured markers of late-life reserve factors, as well as by simultaneously examining the effects of early-, adult-, and, crucially, late-life cognitive reserve factors on dementia risk among the same individuals. Our findings underscore the contribution of factors acting at different periods of the life course.

A marked attenuation in the direct effect of the early-life composite factor on dementia risk could be due to its correlation with the adult-life cognitive reserve-enhancing composite factor. It is noteworthy that the late-life factor was not strongly correlated with either the early- or the adult-life composite factors. One possibility could be that disengagement from late-life leisure activities is a sign of impending dementia, rather than a reflection of earlier-life reserve contributors such as education or occupation. This could also account for the fact that associations between dementia risk and late-life factors were both significant and consistently estimated, whereas the associations between dementia and early-life, as well as adult-life, factors were less precise. Further studies using less correlated indicators are needed to explore the independent effects of early-life influences on dementia risk. Our finding of a dose-response relationship between the cumulative exposure to life-course cognitive reserve-enhancing composite factors and dementia risk underscores the importance of exposures occurring at multiple life periods.

Several biologically plausible hypotheses may explain the association between dementia risk and cognitive reserve factors acting at different periods over the life course. The environment plays a key role in influencing brain plasticity, which is the key element of the brain reserve hypothesis, as well as influencing memory formations and learning processes that provide the brain with a lifelong ability to change and to adjust [50]. Mental stimulation selectively increases synaptogenesis, whereas physical exercise may enhance non-neuronal components of the brain [18]. The ability of the brain to respond to environmental stimuli by adding new neurons or by activating compensatory processes can be sustained in late life [51]. Previous neuroimaging studies have shown that people with higher education, high occupational attainment, or higher levels of intellectual, social, or physical activity may cope with brain damage for a longer period of time [52–55].

We found no evidence of an interaction between APOE ɛ4 status and cognitive reserve factors over the life course on risk of dementia, suggesting that protective effects of cognitive reserve on dementia operate independently of genetic predisposition to the disease, which is consistent with findings from a smaller study using a younger study population [56]. It is known that the structural components of the nervous system are influenced not only by environmental exposures but also as a function of genetic endowment. Although APOE ɛ2 has been consistently shown to have neuroprotective effects and to increase synaptic plasticity [57], APOE ɛ4 has been associated with negative effects on neurites and synaptic functions [58,59]. Reserve-enhancing factors have been shown to enhance the functional organization of the brain through greater resilience in neural circuits involved in cognition and to modify the relationship between senile plaques and cognitive function [4]. Our findings suggest that enhanced neuroplasticity by reserve-enhancing factors may compensate for the deterioration of the brain function to the same extent in both APOE ε4 allele carriers and noncarriers.

Our findings point to the importance of adopting a life-course perspective in designing interventions aimed at enhancing cognitive reserve in order to prevent or postpone dementia incidence. It is never too late to initiate interventions because even late-life activities were associated with lower risk of dementia in our study. Nevertheless, interventions aimed at earlier life periods might be more beneficial, not only because greater exposure frequency has been linked with a reduced risk but also because of the correlated nature of reserve contributors over the life course. Importantly, these interventions should be equally effective among individuals with and without genetic susceptibility.

## Supporting information

S1 Table Risk of dementia in relation to the continuous cognitive reserve latent factors. Adjusted for age, gender, depressive symptoms, comorbidity, and baseline cognitive function.
(DOCX)

S2 Table Total, direct, and indirect effects of cognitive reserve factors on dementia. Results are from Cox models with dementia as the outcome variable. Exposures are the latent factors from early-, adult-, and late-life portions of the life course categorized as dichotomous variables identifying the top three quartiles versus the bottom quartile. First, a full model including all three life-course factors and covariates was fit, with estimated parameters producing the direct effects of early-, adult- and late-life factors. Next, a series of reduced models that included only early-, adult-, or late-life indicators were estimated, with parameters from these models producing the total effect of each life-course indicator. The difference between the total and the direct effect for each of the latent life-course factors yielded an estimate of its indirect effect through all mediating factors. The significance of the indirect effect was tested through the model likelihood ratio, which is −2 times the difference of the log likelihood between the adjacent models, distributed as χ^2^ with the degree of freedom equal to the difference in the number of parameters between the two models. All models are adjusted for age, gender, depressive symptoms, comorbidity, and baseline cognitive function.
(DOCX)

S1 Text SEM computation details. (DOCX)

S2 Text Questions on sibship size, education, occupation, and leisure activities. (DOCX)

## Abbreviations

APOE: apolipoprotein E
MMSE: Mini-Mental State Examination
OR: odds ratio
RR: relative risk
SD: standard deviation
SEM: structural equation model
SES: socioeconomic status
