# Supplementary material for: Association of lifelong exposure to cognitive reserve-enhancing factors with dementia risk: A community-based cohort study
Source: PLoS Med. 2017 Mar 14;14(3):e1002251. doi: 10.1371/journal.pmed.1002251 (PMC5349652; doi:10.1371/journal.pmed.1002251)
Supplement: S1 Table — Adjusted for age, gender, depressive symptoms, comorbidity, and baseline cognitive function. (DOCX) [file pmed.1002251.s001.docx]

**S1 Table.** Risk of dementia in relation to the continuous cognitive reserve latent factors

| Variables | | | Number of. subjects | | Number of cases | RR (95% CI) From separate models adjusted for age and sex | RR (95% CI) From one model with full adjustment* |
| --- | --- | --- | --- | --- | --- | --- | --- |
| **Early life factor** | | |  | |  |  |  |
| Continuous score | 602 | | 148 | 0.61 (0.41-0.90) | 1.19 (0.22-6.39) |  |  |
| **Adulthood factor** | |  |  |  |  |  |  |
| Continuous score | 602 | | 148 | 0.53 (0.31-0.91) | 0.57 (0.06-5.26) |  |  |
| **Late life factor** | | |  | |  |  |  |
| Continuous score | 602 | | 148 | 0.70 (0.53-0.92) | 0.80 (0.58-1.09) |  |  |

Adjusted for age, gender, depressive symptoms, co-morbidity, and baseline cognitive function.
