# Supplementary material for: Association of lifelong exposure to cognitive reserve-enhancing factors with dementia risk: A community-based cohort study
Source: PLoS Med. 2017 Mar 14;14(3):e1002251. doi: 10.1371/journal.pmed.1002251 (PMC5349652; doi:10.1371/journal.pmed.1002251)
Supplement: S2 Table — Results are from Cox models with dementia as the outcome variable. Exposures are the latent factors from early-, adult-, and late-life portions of the life course categorized as dichotomous variables identifying the top three quartiles versus the bottom quartile. First, a full model including all three life-course factors and covariates was fit, with estimated parameters producing the direct effects of early-, adult- and late-life factors. Next, a series of reduced models that included only early-, adult-, or late-life indicators were estimated, with parameters from these models producing the total effect of each life-course indicator. The difference between the total and the direct effect for each of the latent life-course factors yielded an estimate of its indirect effect through all mediating factors. The significance of the indirect effect was tested through the model likelihood ratio, which is −2 times the difference of the log likelihood between the adjacent models, distributed as χ2 with the degree of freedom equal to the difference in the number of parameters between the two models. All models are adjusted for age, gender, depressive symptoms, comorbidity, and baseline cognitive function. (DOCX) [file pmed.1002251.s002.docx]

**S2 Table.** Total, direct and indirect effects of cognitive reserve latent factors on dementia

| Type of effect | Effect estimate  (b-coefficient) | Percent of effect | p-value |  |
| --- | --- | --- | --- | --- |
| Direct effect of early life | | -.278 | 60.7 |  |
| Indirect effect | | -.180 | 39.3 | <0.001 |
| Through midlife | | -.096 | 21.0 | <0.01 |
| Through late life | | -.084 | 18.3 | <0.05 |
| Total effect of early life | | -.458 |  |  |
| Direct effect of midlife | | -.317 | 85.4 |  |
| Indirect effect through late life | | -.054 | 14.6 | <0.001 |
| Total effect of midlife | | -.371 |  |  |
| Direct effect of late life | | -.426 |  |  |

Results are from Cox models with dementia as the outcome variable. Exposures are the latent factors from early-, adult-, and late-life portions of the life course categorized as dichotomous variables identifying the top three quartiles vs. the bottom quartile. First, a full model including all three life course factors and covariates was fit, with estimated parameters producing the direct effects of early, adult- and late-life factors. Next, a series of reduced models that included only early-, adult-, or late life indicators were estimated, with parameters from these models producing the total effect of each life course indicator. The difference between the total and the direct effect for each of the latent life course factors yielded an estimate of its indirect effect through all mediating factors. The significance of the indirect effect was tested through the model likelihood ratio, which is -2 times the difference of the log likelihood between the adjacent models, distributed as χ2 with degree of freedom equal to the difference in the number of parameters between the two models. All models are adjusted for age, gender, depressive symptoms, co-morbidity, and baseline cognitive function.
