# Supplementary material for: Association of lifelong exposure to cognitive reserve-enhancing factors with dementia risk: A community-based cohort study
Source: PLoS Med. 2017 Mar 14;14(3):e1002251. doi: 10.1371/journal.pmed.1002251 (PMC5349652; doi:10.1371/journal.pmed.1002251)
Supplement: S1 Text — (DOCX) [file pmed.1002251.s003.docx]

**SEM Computation Details.** Structural Equation Modeling was used to derive a best-fitting measurement model for ten individual life-course indicators. This model emphasizes construct variance associated with specific life-course indicators (e.g., education, occupational complexity, leisure activities), minimizes variance specific to particular procedures or stimulus materials, and reduces the number of indicators entered into the survival models. A best-fitting latent measurement model for the three life-course factors (early-, adult-, and later-life) was obtained using the AMOS 24 software package. The model was scaled by fixing a single indicator measure to 1 for each factor. Model fit was evaluated using various fit criteria including the chi-square (χ2) goodness of fit test, the Comparative Fit Index (CFI), the Goodness of Fit Index (GFI), the Root Mean Square Error of Approximation (RMSEA), and the ratio of χ2 to degrees of freedom. According to conventions associated with these criteria, the overall fit of the 3-factor measurement model was good: χ2 = 99.81, df = 31, p = 0.000, χ2/df ratio = 3.22, CFI = 0.901, GFI = 0.967 and RMSEA = 0.061. Factor-score weights were derived from the SEM model and used to calculate composite latent factors for each portion of the life course. Specifically, for each latent factor, an individual’s factor score was estimated by: 1) standardizing measurements on the raw indicators; 2) multiplying the standardized score for each indicator by its corresponding SEM factor-score weight; 3) summing the products to yield three separate latent factor-score estimates for the early-, adult-, and late-life periods. The distribution of the latent variables is as follows: early life (range from -1 to 1, Mean=0.02, SD=0.491), adult life (range from -1 to 1, Mean=0.02, SD=0.372) and late life (range from -1 to 1, Mean=0.10, SD=0.655). The RR’s from models with latent variables should be interpreted as per 1 unit SD increases in the underlying latent factor. The correlation between the early- and the adult-life latent factors was strong (γ=0.9).
