# Supplementary material for: Association of lifelong exposure to cognitive reserve-enhancing factors with dementia risk: A community-based cohort study
Source: PLoS Med. 2017 Mar 14;14(3):e1002251. doi: 10.1371/journal.pmed.1002251 (PMC5349652; doi:10.1371/journal.pmed.1002251)
Supplement: S2 Text — (DOCX) [file pmed.1002251.s004.docx]

Questions on sibship size, education, occupation, and leisure activities administered during nurse interviews at study baseline and first follow-up examination. Kungsholmen Project ^[[1]](#footnote-1)^

1. Did you have any older or younger brothers or sisters growing up with you?
   1. Yes
   2. No
   3. No answer
2. If yes, how many siblings did you have?
   1. __________
3. Which of these types of schooling corresponds to your longest-attended education?
   1. Pre-elementary school (1-2 school years)
   2. Elementary school (3-7 school years)
   3. Secondary school (9 school years)
   4. High school (12 school years)
   5. University (15-20 school years)
   6. Other – specify
   7. No answer
4. Have you ever been working?
5. Yes
6. No
7. No answer
8. If yes, what has been your main occupation?
9. __________
10. Please name 4 main occupations you held at different stages during your life.
11. Describe the nature of work conducted for each position.
12. Indicate the starting and finishing years for each position.
    - 1. Starts from year _____ Ends in year________
13. Are you still working?
14. Yes
15. No
16. No answer
17. Describe the nature of the work you currently perform
18. _____________
19. How much time do you spend in current work?
    1. _______ Hours per week.
20. Do you currently engage in regular leisure activities?
    1. Yes
    2. No
    3. No answer
21. List all leisure activities you currently engage in.
    1. ________

1. Questions are translated from Swedish. Questions on sibship size were administered during the nurse interview at study baseline (1987-1989) and were answered by the study participants themselves. Educational and leisure questionnaire was administered at the same nurse interview at baseline and was answered by the study participants themselves. Occupational questionnaire was administered at the first follow-up examination (2-3 years after baseline assessment) and was answered by informants for both study participants with dementia and dementia-free individuals. [↑](#footnote-ref-1)
